# Therapeutic effect of turmeric on radiodermatitis: A systematic review

**DOI:** 10.14814/phy2.15624

**Published:** 2023-03-05

**Authors:** Atieh Ostadi, Morteza Arab‐Zozani, Elham Zarei, Gordon A. Ferns, Afsane Bahrami

**Affiliations:** ^1^ Faculty of Paramedical, Mashhad Branch Islamic Azad University Mashhad Iran; ^2^ Social Determinants of Health Research Center Birjand University of Medical Sciences Birjand Iran; ^3^ Mashhad University of Medical Sciences Mashhad Iran; ^4^ Brighton & Sussex Medical School Division of Medical Education Falmer UK; ^5^ Clinical Research Development Unit Imam Reza Hospital, Faculty of Medicine Mashhad University of Medical Sciences Mashhad Iran; ^6^ Clinical Research Development Unit of Akbar Hospital Faculty of Medicine, Mashhad University of Medical Sciences Mashhad Iran

**Keywords:** curcuma, Radiodermatitis, radiotherapy, turmeric

## Abstract

Radiodermatitis (RD) occurs in 95% of cancer patients undergoing radiation therapy. At present, there is no effective treatment for the management of this complication of radiotherapy. Turmeric (*Curcuma longa*) is a polyphenolic and biologically active natural compound with various pharmacological functions. The aim of this systematic review was to determine the efficacy of curcumin supplementation for reducing RD severity. This review complied with the Preferred Reporting Items for Systematic Reviews and Meta‐Analyses (PRISMA) statement. A comprehensive literature search was conducted in Cochrane library, PubMed, Scopus, Web of Science, and MEDLINE databases. A total of seven studies comprising 473 cases and 552 controls were included in this review. Four studies demonstrated that curcumin supplementation had a beneficial effect on RD intensity. These data provide evidence for the potential clinical use of curcumin in supportive cancer care. Further large prospective and well‐designed trials are warranted to exactly determine the “real effective extract, supplemental form and dose of curcumin” for RD prevention and treatment of patients receiving radiotherapy.

## Graphical abstract

Radiodermatitis (RD) occurs in 95% of cancer patients undergoing radiation therapy. At present, there is no effective treatment for the management of this complication of RD. Turmeric (*Curcuma longa*) is a polyphenolic and biologically active natural compound with various pharmacological functions. The aim of this systematic review and meta‐analysis was to determine the efficacy of curcumin supplementation at reducing RD severity. Most of the studies demonstrated that curcumin supplementation had a beneficial effect on RD intensity. These data provide strong evidence of curcumin's clinical value in supportive cancer care.

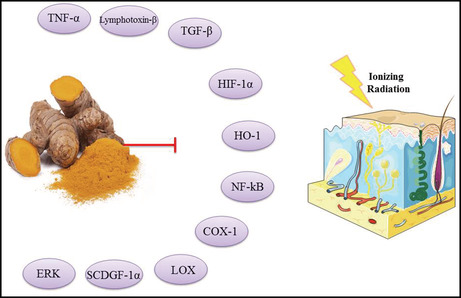

## 1 BACKGROUND

Radiodermatitis (RD) is one of the most frequent adverse consequences experienced by patients undergoing radiotherapy (RT) for different cancers (Ryan et al., 2013). RD is due to DNA damage and modification of proteins, lipids, or carbohydrates that lead to destruction and injury of the dermal basal cells (Zhang et al., 2013). The clinical manifestations of RD vary, but may include: feeble erythema, dry desquamation, clammy desquamation, pigmentation alterations, telangiectasias, hair loss, necrosis, fibrosis and demolition of the adnexa, and deep ulcerations (McQuestion, 2006). Acute RD may affect quality of life and in some patients leads to unplanned gaps during treatment and interrupt treatment protocols (Bataini et al., 1988).

The management of severe RD is important in the treatment of patients with curative RT. Except for skin cleaning with lukewarm water and lenient soap as well as inhibition of local trauma, there is no established protocol for ameliorating skin radiation toxicity (Campbell & Illingworth, 1992; Lavery, 1995; Roy et al., 2001). Over the last decade, the effects of a wide range of treatments have been suggested for this condition, consisting of moisturizers, anti‐inflammatory agents, *Aloe vera* gel, fatty ointments, silymarin and marigold, curcumin (CUM), and different creams (chamomile, almond, steroid and non‐steroid) (Falkowski et al., 2011; Heggie et al., 2002; Pommier et al., 2004).

Various investigations have been performed to assess the efficiency of topical compounds on RD. The findings of a systematic review summarizing five studies demonstrated that deodorant or antiperspirant had no effect on development of RD. Nonsteroidal topical agents had a small positive effect on the incidence of moistdesquamation and treatment of itching (Ginex, 2020). Results from a meta‐analysis conducted in 2013 showed that corticosteroids and other conventional topical agents including trolamine, sucralfate, gentian violet, *Aloe vera*, urea, biafine, mixture of oil and aqueous, vitamin C, and hyaluronic acid were unsuccessful at effective prevention and treatment of RD (Zhang et al., 2013).

CUM (1,7‐bis(4‐hydroxy‐3‐methoxyphenyl)1,6‐heptadiene‐3,5‐dione) is a yellow colored polyphenol, low‐molecular‐weight natural polyphenol derived from a perennial herb, turmeric (*Curcuma longa L*), that has low intrinsic toxicity in humans (Hosseini et al., 2017). CUM has been found to possess a range of pharmacological functions including anti‐microbial effects, anti‐inflammatory, anti‐oxidant, anti‐cancer, and chemotherapeutic properties (Sahebkar, 2014; Tajbakhsh et al., 2018). The remarkable effects of CUM include the functional and gnomic suppression of enzymes involved in producing ROS and inflammatory lipids, pro‐inflammatory transcription factors, also over‐expression of anti‐oxidant pathways (Ryan et al., 2013).

CUM derivatives have been found to inhibit amyloid formation during wound healing and has been utilized to treat skin disorders, such as eczema, scabies, acne, and crease skin, as well as preventing chemical and UV‐induced skin tumorgenesis in animal studies (Dwivedi & Abu‐Ghazaleh, 1997; Dwivedi et al., 2003, 2005). Some studies have also reported the wound healing effect of CUM in combination with other agent, such as ginger and *Aloe vera* (Bhagavathula et al., 2009; Fray et al., 2004). Results from a recent systematic review has claimed that CUM may have preventive and therapeutic effect on skin redness, transepidermal water loss, and lesions created by RD, psoriasis, and Bowen disease (Barbalho et al., 2021). However, the therapeutic potential of CUR in human studies have been inconsistent (Palatty et al., 2014; Wolf et al., 2018).

This review summarized the available evidence support the efficiency of CUM at preventing and treating RD. Estimates of the importance of CUM treatment could improve clinical management, and would assist clinicians to choose better therapeutic strategies for patients.

## 2 METHODS

### 2.1 Searches

We used multiple databases to identify human intervention studies on the effect of purified CUR or curcuminoids mixture, or standardized *Curcuma* spp. extracts on RD following Preferred Reporting Items for Systematic Reviews and Meta‐analyses (PRISMA) statements (File S1).

**FIGURE 1:**
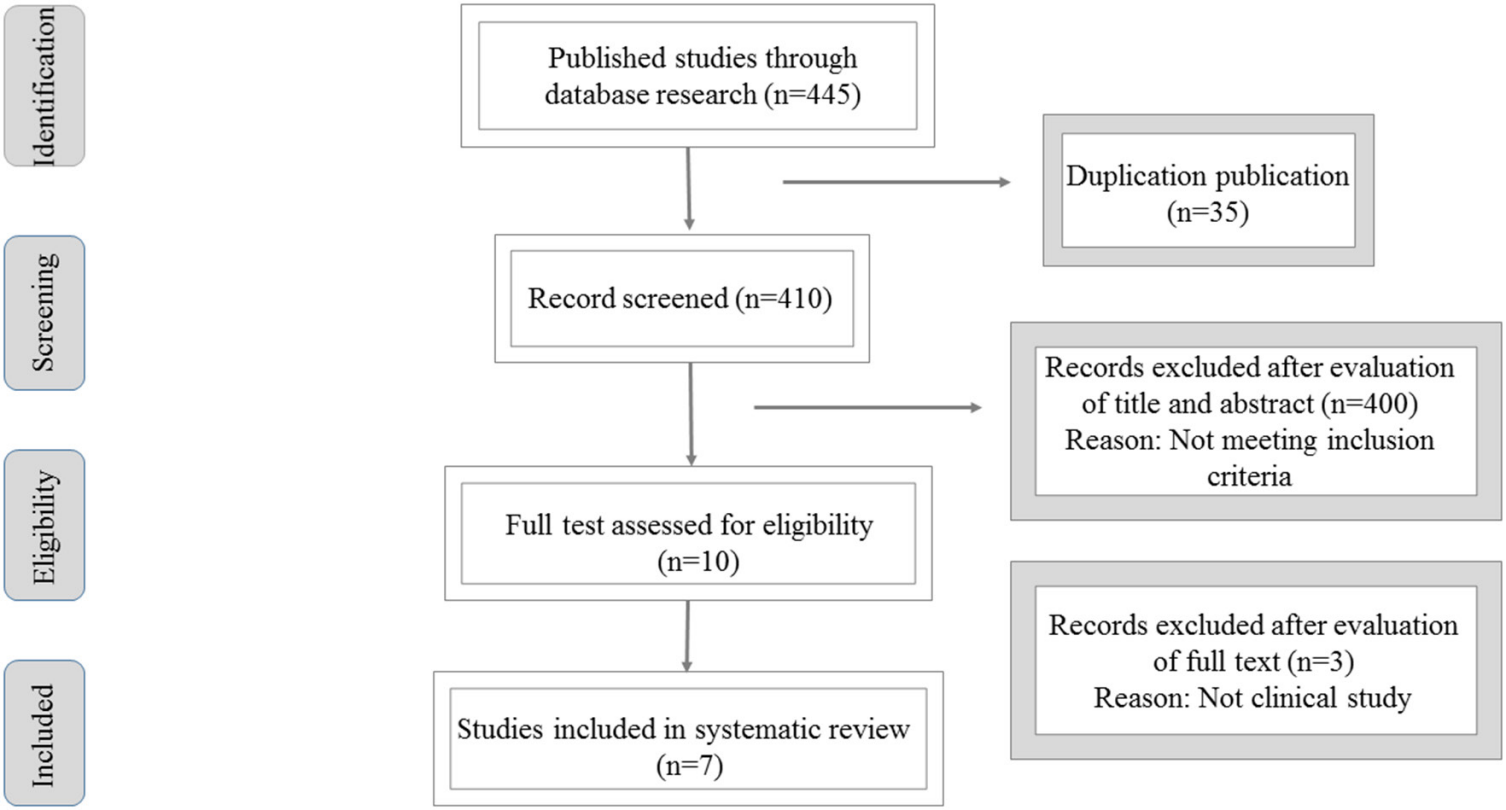
Flowchart of literature search and selection process.

A systematic literature search was performed in Cochrane library, PubMed, Scopus, Web of Science, and MEDLINE limited to English language and published from 1950 to the end of June, 2022. The following keywords were applied for the search separately or together: “curcumin”, “*curcuma”*, “turmeric”, “curcuma domestica”, “*Curcuma Longa L”*, “radiotherapy”, “dermatitis”, “radiodermatitis” and “radiation dermatitis”. Details of the literature search were shown in Figure 1. Also, reference lists of selected articles and other relevant papers were manually searched. Overlapping records were considered just once.

The inclusion criteria were (i) study searched on cancer patients undergoing RT and (ii) evaluated the effect of CUR on RD severity. Any irrelevant records, review articles, meeting abstracts, case reports, in vitro data, and animal studies were excluded. Screening of eligible records and selection of articles to be entered were independently carried out by two expert reviewers and any disagreements in opinion were resolved by consensus with supervisors.

### 2.2 Data extraction

For each selected study, the following information was retrieved: authors, year, location, design, cancer type, mean receiving radiation dose, number of participants, dose, and duration of intervention as well as assessed measurements and main finding.

### 2.3 Risk of bias in individual studies

Two reviewers (A.B. and M.A.‐Z.) independently assessed the risk of bias among the included studies. We used the Cochrane Collaboration's tool for assessing risk of bias in the included randomized trials, including seven items of selection bias (random sequence generation and allocation concealment), performance bias, detection bias, attrition bias, reporting bias, and other forms of bias. Any disagreements were resolved upon consultation with research team.

## 3 RESULTS

### 3.1 Results of the literature search

According to the search strategy, 438 papers were identified, of which 91% were excluded after removing duplicates and evaluating the titles and abstracts. After assessing the full text of the remaining nine articles, seven articles were selected for comprehensive review (Figure 1).

### 3.2 Characteristics of included articles

Finally, seven studies that comprised 473 cases and 552 controls included in this review.

Among the seven selected records, were randomized clinical trials (RCT) (Rao et al., 2017; Ryan et al., 2013; Ryan Wolf et al., 2020; Wolf et al., 2018) and three retrospective, pilot, and controlled study designed, respectively (Manjunatha et al., 2020, Palatty et al., 2014, Belcaro et al., 2014). Four studies conducted among breast cancer (BC) patients (Rao et al., 2017, Ryan et al., 2013, Ryan Wolf et al., 2020, Wolf et al., 2018), but one enrolled head and neck cancer patients (Palatty et al., 2014), one cervical cancer patients (Manjunatha et al., 2020), and the other carried out study among patient with different cancers (tumors of colon/rectum, liver, kidney, stomach, lung, and ovary or hematological malignancies) (Belcaro et al., 2014).

The duration of intervention in these studies was between 5 and 12 weeks. A wide range of mean radiation dosing (30–66 Gy) was undertaken for RT in the selected articles. Four studies used oral supplements (Belcaro et al., 2014; Manjunatha et al., 2020; Ryan et al., 2013; Ryan Wolf et al., 2018) and three studies provided topical numeric creams (Palatty et al., 2014; Rao et al., 2017; Ryan Wolf et al., 2020) (Table 1). Only two study used purified CUM for supplementation (Ryan et al., 2013, Manjunatha et al., 2020), while two studies used curcuminoids (contain CUM, dimethoxy CUM, and bisdemethoxy CUM) (Belcaro et al., 2014; Wolf et al., 2018), one study administrated curcumin gel (Ryan Wolf et al., 2020), and the remaining studies utilized Vicco turmeric cream (VTC), consisting of turmeric and sandal wood oil (*Santalum album L*) (Palatty et al., 2014; Rao et al., 2017). Four articles administrated placebo (Belcaro et al., 2014; Ryan et al., 2013; Ryan Wolf et al., 2020; Wolf et al., 2018), three studies prescribed moisturizing cream or Johnsons Baby Oil (JBO) (Palatty et al., 2014; Rao et al., 2017; Ryan Wolf et al., 2020), though only included a group under observation as a comparator group.

**TABLE 1:** Characteristics of included studies.

Author, year	Ryan et al. (2013)	Palatty et al. (2014)	Belcaro et al. (2014)	Rao et al. (2017)	Ryan Wolf et al. (2018)	Ryan Wolf et al. (2020)	Manjunatha et al. (2020)
Country	USA	India	Italy	India	USA	USA	India
Design	Double blind RCT	Pilot	Controlled study	Investigator‐blinded RCT	Double‐blinded RCT	Three‐arm‐semi‐blind RCT	Retrospective analysis
Duration of trial	7 weeks	7 weeks	60 days	5 weeks	During course of RT until 1 week post RT	During course of RT until 1 week post RT	A minimum period of 3 months post RT
Type of cancer	Breast	Head/neck	All types	Breast	Breast	Breast	Cervix
Intervention							
Case	Curcumin (2.0 g, three times daily)	VTC(2 g, times times daily)	Curcuminoids (100 mg, three times daily)	VTC(5 gr, five times daily)	Curcuminoids (2.0 gr, three times daily)	Psoria‐Gold® Curcumin gel (three times daily)	Tab. Curcumin 500 mg BID
Control	Placebo	Moisturizing cream, JBO (2 mL, daily)	Placebo	Moisturizing cream, JBO (5 mL, five times daily)	‐Placebo	HPR Plus™	Only under observation
						Placebo gel	
Sample size							
Case	14	22	40	20	283	64	30
Control	16	24	40	20	295	65	30
						62	
Mean radiation dose (Gy)	46.51 ± 3.48	66.0 ± 5.70	30–50	50	48.34 ± 0.14	44–66	45–50
Assessed measurements	RDS score	Signs of skin damage based on the criteria of (RTOG/EORTC)	The incidence of side effects (damage to epithelial surfaces) PFR status	Dermatological analysis based on the criteria of (RTOG/EORTC)	RDS score Pain symptoms Quality of life	RDS score Pain symptoms	Occurrence of RD Cystitis Proctitis
Main result	Curcumin decreased RDS score compared to placebo (2.6 vs. 3.4) Curcumin group had lower moist desquamation (28.6% vs. 87.5%) No significant difference was found in radiation skin dose, redness, associated pain or symptoms between groups	Significant decrement of RD grades in VTC group at all timepoints Occurrence of grade 3 RD was lower in the VTC arm Significant reduced degree of RD 2 weeks after the completion of RT in VTC arm compared to JBO group (0.82 vs.1.3)	Significant lower incidence of skin damage in curcuminoids compared to placebo groups (22% vs. 51%) Decrement in PFR levels in curcumin, while an increment in PFR in the control group	Significant decrement in the occurrence of grade 1 (at week two) and grade 2 and 3 (at week 3 and 4) in the VTC arm versus JBO	No significant difference was found in RDS score, pain, symptoms, and quality of life between arms	No significant difference was found in RDS score between arms. Curcumin minimize RDS score and pain for patients with high breast separation	Significant lower incidence of RD (40% vs 67%), cystitis 40% vs. 70%) and proctitis (30% vs 77%) in cucumin compared to observation group

Abbreviations: JBO, Johnsons Baby Oil; PFR, plasma‐free radical; RDS, Radiation Dermatitis Severity; RT, radiation therapy; RTOG/EORTC, Radiation Therapy Oncology Group/European Organization for Research and Treatment Cancer; VTC, Vicco Turmeric Cream.

### 3.3 Outcome

Rao and colleagues compared the preventive effect of VTC (5 g/five times daily) on RD grades with JBO (5 mL/five times daily) as placebo in BC patients under RT. The results indicated that the topical application of VTC delayed and reduced the intensity of RD during the 5 week intervention (Rao et al., 2017). In an exploration on 50 patients with head and neck malignancy, a significant attenuation in grades of RD were observed in patients using tropical VTC (2 g/five times per day) at all timepoints, including 2 weeks after RD completion versus those received JBO (2 mL/five times per day). Furthermore, the incidence of grade 3 RD was significantly lower in the VTC group (Palatty et al., 2014).

Results from double‐blinded RCT of 686 BC patients demonstrated that CUM (2.0 g capsules of curcuminoids; three times per day) did not reduce the RDS score, associated pain and symptoms as well as quality of life of patients at the end of trial compared to placebo (Ryan Wolf et al., 2018). Ryan Wolf et al. was also performed a semi‐blinded RCT among 171 BC patients to compare the prophylactic potency of three topical‐use compounds (CUM, HPR Plus™, and placebo) for reducing RD pain. HPR Plus™ is an FDA‐approved medical moisturizer indicated for different types of dermatoses and RD. Mean RDS scales did not significantly differ between study groups (CUM = 2.68; HPR Plus™ = 2.64; placebo = 2.63; *p* = 0.929) at the end of RCT. But subgroup analysis indicates that CUM therapy may be more effective for reducing score of RDS and pain in patients with high breast separation (≥25 cm) who may have the severe RD (Ryan Wolf et al., 2020). Ryan et al. conducted a double‐blind, RCT to evaluate the therapeutic potency of oral CUM (6.0 g daily) to ameliorate the RDS in 30 BC patients undergoing RT without chemotherapy. Analysis demonstrated that CUM reduced RDS score and incidence of moist desquamation versus placebo within radiation. Although, no significant differences were found between the study groups regarding to the redness, associated pain, or symptoms (Ryan et al., 2013).

In another study, 60 cervical cancer patients who received chemoradiotherapy were included for the investigation. Of these, 30 patients received CUM tablets (500 mg BID) for at least 3 months post RT during follow‐up and were compared with cases of similar features kept only under observation. Occurrence of RD was significantly lower in CUM arm compared to observation groups (40% vs. 67%; *p* = 0.038) (Manjunatha et al., 2020).

Belcaro et al. studied the effect of Meriva tablets, a lecithinized formulation of CUM, on side effects of RT among 160 cancer patients. Patients administrated to receive 500 mg Meriva (consist of curcuminoids, soy lecithin and microcrystalline cellulose; ratio 1: 2: 2) or placebo. After 60 days, there was a significantly lower incidence of skin damage was found in curcuminoids arm compared to placebo group (22% vs. 51%, *p* < 0.05). Meriva also did not show any significant adverse effect, with good tolerability and high compliance (Belcaro et al., 2014).

### 3.4 Quality appraisal

**FIGURE 2:**
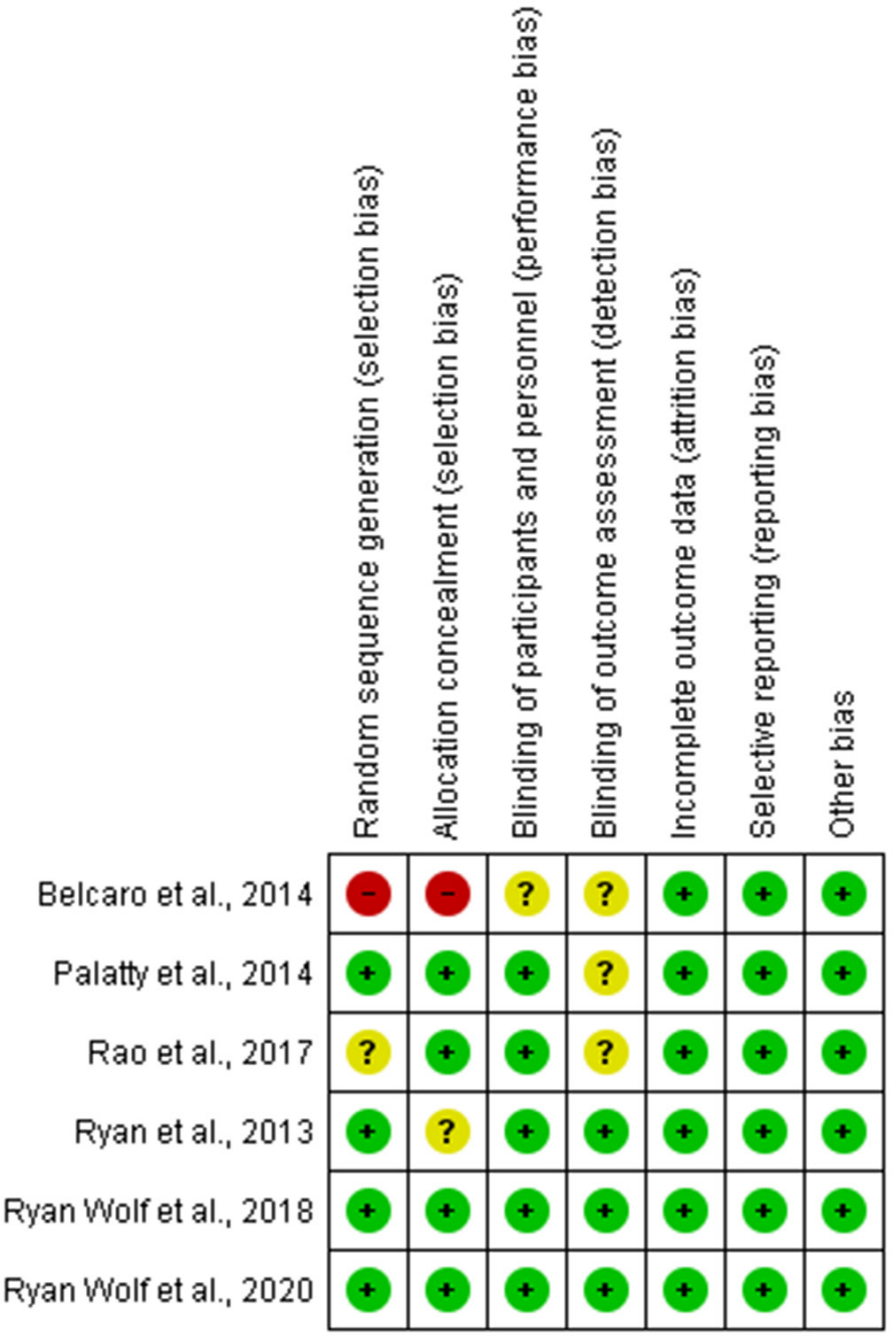
Risk of bias graph: review authors' judgments about each risk of bias item presented as percentages across all included studies.

**FIGURE 3:**
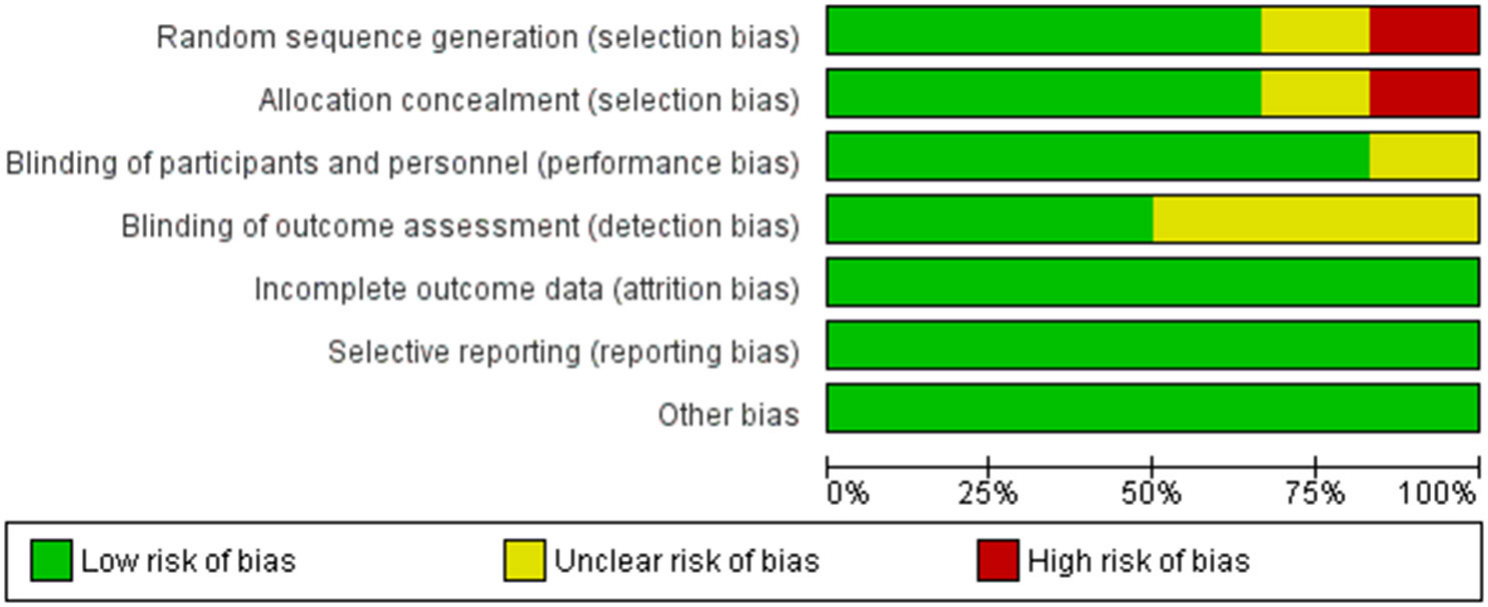
Risk of bias summary: review authors' judgments about each risk of bias item for each included study.

Six of the included studies were randomized trial. Four (66.6%) studies described the random sequence generation and the allocation concealment in an acceptable manner. Five studies reported acceptable blinding for participants and personnel. Three studies (50%) reported blinding of outcome assessment. The risk of bias summary and risk of bias graph is reported in Figures 2 and 3.

## 4 DISCUSSION

Findings from the present review provides evidence for the beneficial effects of CUM on improving RD in patients with cancer receiving RT (Table 1). Preclinical studies in experimental animal models have shown that CUM supplementation was potentially effective in wound healing in radiation‐exposed mice. The process of RD leads to production of free radicals and DNA damages (Schaue et al., 2012; Stone et al., 2003). There is a large body of evidence that indicates that CUM possesses free radical scavenging activity of reactive oxygen and nitrogen species (Baliga et al., 2013; Gupta et al., 2013). Also, results from in vivo and in vitro studies supported CUMs antioxidants functions and prevention of lipid peroxidation and DNA strands break (Jelveh et al., 2013; Parshad et al., 1998). CUM promoted the rate of wound contraction and re‐epithelialization of the epidermis, reduced mean healing time, incremental neovascularization, and elevated generation and deposition of collagen at site of injury (Jagetia & Rajanikant, 2004, 2005; López‐Jornet et al., 2011). CUM also protect against ionizing irradiation‐associated cataractogenesis in rats (Özgen et al., 2012). Vaughn et al. undertook a systematic review of the effect of both topical and oral CUM on skin health in different disorders such as acne, atopic dermatitis, alopecia, facial photoaging, pruritus, and psoriasis. Of the 18 studies, 10 of them highlighted the ameliorating effect of turmeric in skin disease severity (Vaughn et al., 2016).

Moreover, CUM elicits anti‐inflammatory effects against inflammatory mediators in cutaneous tissues (Huang et al., 1997) by inhibiting ornithine decarboxylase responses, DNA synthesis, epidermal lipoxygenase and cyclooxygenase (COX) activity, and activation of inflammatory pathways including extracellular signal‐related kinase (ERK), and nuclear factor kB (NF‐kB) signaling pathways in stimulated cells (Baliga et al., 2013; Chun et al., 2003; Gupta et al., 2013; Thangapazham et al., 2013). Furthermore, CUM significantly decreases acute and chronic skin toxicity in mice and exerts protective effects by downregulating early reacting cytokines and interleukins including tumor necrosis factor α (TNF‐α), lymphotoxin‐β, TGF‐β, hypoxia‐inducible growth factor‐1α, stromal cell‐derived growth factor‐1α, and hemeoxygenase‐1 in epidermis (Okunieff et al., 2006). A hot water extract of *Curcuma longa* has anti‐inflammatory effects by suppressing the elevation in ultraviolet B‐induced TNF‐α and IL‐1β as well as significant enhancing hyaluronan synthesis from non‐stimulated keratinocytes (Asada et al., 2019).

Despite its great biological effects, oral CUM administration (up to 8 g/day) has low bioavailability and absorption rate in the gastrointestinal tract, as well as rapid metabolism and elimination from the body. Previously, it has been shown that CUM gel was biologically acceptable to all individuals without any adverse effects or allergic reactions (Anuradha et al., 2015). Only, three studies investigated the effect of turmeric cream or CUM gel on RD, which advantageous effects imply important therapeutic implications. Local delivery provided higher amounts of the active ingredients in the diseased region and prevents probable side effects of systemic administration. Easy to use, non‐greasy, patient adherence, prolonged residence duration on the skin, and superior drug release are benefits of topical application. However, topical administration has also been restricted due to the vibrant yellow color pigment of turmeric which is part of the active CUM component, since it may stain the skin.

The small number of clinical trials included may be regarded as a main limitation of the current review of the potential beneficial effects of CUM treatment on RD severity in human cancer patients. Furthermore, included studies were heterogeneous due to their study designs, baseline demographic characteristics, tumor types, mean received radiation dose, supplement form, dose, and duration.

## 5 CONCLUSIONS

This review provides evidence on the potential clinical value of CUM in cancer supportive care. There was no significant difference in outcomes between topical or oral administration. With respect to the prior equivocal findings and that three out the seven studies in the present systematic review did not show a significant benefit of CUM treatment, our findings should be interpreted with caution. An objective scale for RD severity and further investigation for an efficient therapy for RD is needed. Further prospective large and well‐designed trials are warranted to exactly determine the “real effective extract, supplemental form and dose of curcumin” for RD prevention and treatment in patients receiving RT.

## AUTHOR CONTRIBUTIONS

AB: Conceptualization. AO, MA, EZ: Data curation. AB: Investigation. AO, EZ, MA, GF: Methodology. AB: Project administration. AB: Supervision. GF: Validation. AB: Visualization. AO, AB: Writing (original), draft preparation. GF: Writing–review and editing. All authors have read and approved the final submitted manuscript.

## CONFLICT OF INTEREST STATEMENT

The authors declare no conflict of interest.

## ETHICAL APPROVAL

Not applicable.

## Supporting information


Supporting information S1.
